# The impact of p53 on DNA damage and metabolic activation of the environmental carcinogen benzo[*a*]pyrene: effects in *Trp53(*+*/*+*)*, *Trp53(*+*/*–*)* and *Trp53(*−*/*−*)* mice

**DOI:** 10.1007/s00204-015-1531-8

**Published:** 2015-05-21

**Authors:** Annette M. Krais, Ewoud N. Speksnijder, Joost P. M. Melis, Radek Indra, Michaela Moserova, Roger W. Godschalk, Frederik-J. van Schooten, Albrecht Seidel, Klaus Kopka, Heinz H. Schmeiser, Marie Stiborova, David H. Phillips, Mirjam Luijten, Volker M. Arlt

**Affiliations:** Analytical and Environmental Sciences Division, MRC-PHE Centre for Environment & Health, King’s College London, Franklin-Wilkins Building, 150 Stamford Street, London, SE1 9NH UK; Center for Health Protection, National Institute for Public Health and the Environment (RIVM), 3721 MA Bilthoven, The Netherlands; Department of Human Genetics, Leiden University Medical Center, 2300 RC Leiden, The Netherlands; Department of Biochemistry, Faculty of Science, Charles University, 12840 Prague 2, Czech Republic; Department of Toxicology, School for Nutrition, Toxicology and Metabolism (NUTRIM), Maastricht University Medical Centre, 6200 MD Maastricht, The Netherlands; Biochemical Institute for Environmental Carcinogens, Prof. Dr. Gernot Grimmer-Foundation, 22927 Grosshansdorf, Germany; Division of Radiopharmaceutical Chemistry, German Cancer Research Center (DKFZ), Im Neuenheimer Feld 280, 69120 Heidelberg, Germany

**Keywords:** Benzo[*a*]pyrene, Tumour suppressor p53, Mouse models, Cytochrome P450, Carcinogen metabolism, DNA adducts

## Abstract

The tumour suppressor p53 is one of the most important cancer genes. Previous findings have shown that p53 expression can influence DNA adduct formation of the environmental carcinogen benzo[*a*]pyrene (BaP) in human cells, indicating a role for p53 in the cytochrome P450 (CYP) 1A1-mediated biotransformation of BaP in vitro. We investigated the potential role of p53 in xenobiotic metabolism in vivo by treating *Trp53(*+*/*+*)*, *Trp53(*+*/*–*)* and *Trp53(*−*/*−*)* mice with BaP. BaP-DNA adduct levels, as measured by ^32^P-postlabelling analysis, were significantly higher in liver and kidney of *Trp53(*−*/*−*)* mice than of *Trp53(*+*/*+*)* mice. Complementarily, significantly higher amounts of BaP metabolites were also formed ex vivo in hepatic microsomes from BaP-pretreated *Trp53(*−*/*−*)* mice. Bypass of the need for metabolic activation by treating mice with BaP-7,8-dihydrodiol-9,10-epoxide resulted in similar adduct levels in liver and kidney in all mouse lines, confirming that the influence of p53 is on the biotransformation of the parent compound. Higher BaP-DNA adduct levels in the livers of *Trp53(*−*/*−*)* mice correlated with higher CYP1A protein levels and increased CYP1A enzyme activity in these animals. Our study demonstrates a role for p53 in the metabolism of BaP in vivo, confirming previous in vitro results on a novel role for p53 in CYP1A1-mediated BaP metabolism. However, our results also suggest that the mechanisms involved in the altered expression and activity of the CYP1A1 enzyme by p53 in vitro and in vivo are different.

**Electronic supplementary material:**

The online version of this article (doi:10.1007/s00204-015-1531-8) contains supplementary material, which is available to authorized users.

## Introduction

The *TP53* tumour suppressor gene, which encodes the protein p53, is often described as the guardian of the genome and is the most commonly mutated gene in human tumours (Olivier et al. 2010). As gatekeeper, p53 regulates cell growth by inhibiting proliferation or promoting apoptosis (Taneja et al. 2011). As caretaker, it controls cellular processes to maintain genomic integrity, including repair to remove DNA damage (Taneja et al. 2011). Disruption of the normal p53 response by *TP53* mutation leads to increased risks of tumour development. *TP53* is mutated in over 50 % of sporadic tumours, and various environmental carcinogens have been found to be associated with characteristic mutational signatures in *TP53* (Olivier et al. 2010). In addition to somatic mutations in the *TP53* gene, germline mutations have been found to cause predisposition to cancer, and *TP53* polymorphisms have been shown to increase cancer susceptibility (Whibley et al. 2009). Besides its role in DNA damage response, p53 has also been found to regulate metabolic pathways, thereby linking p53 not only to cancer, but also to other diseases such as diabetes and obesity (Maddocks and Vousden 2011).

Previously, we used a panel of isogenic human colorectal carcinoma HCT116 cell lines that differed only with respect to their endogenous *TP53* status in order to investigate the metabolism and DNA damage induced by the environmental carcinogens benzo[*a*]pyrene (BaP) and 3-nitrobenzanthrone (3-NBA) (Hockley et al. 2008; Wohak et al. 2014). We found that HCT116 *TP53(*−*/*−*)* and *TP53(*+*/*–*)* cells formed significantly lower BaP-DNA adduct levels than *TP53(*+*/*+*)* cells. In contrast, no difference in adduct formation was observed in HCT116 cells exposed to BaP-7,8-diol-9,10-epoxide (BPDE), the activated metabolite of BaP, indicating that p53 expression is linked to the cytochrome P450 (CYP)-mediated metabolic activation of BaP (compare Supporting Figure 1a). There were also significantly lower levels of BaP metabolites detected in the culture media of HCT116 *TP53(*−*/*−*)* and *TP53(*+*/*–*)* cells relative to *TP53(*+*/*+*)* cells, which was accompanied by a greater induction of CYP1A1 protein and *CYP1A1* mRNA in *TP53(*+*/*+*)* cells than in the other cell lines (Wohak et al. 2014). We found that BaP-induced CYP1A1 expression was regulated through a p53 response element (p53RE) in the regulatory region of *CYP1A1*, thereby providing a novel pathway for the induction of CYP1A1 by polycyclic aromatic hydrocarbons (PAHs) such as BaP (Wohak et al. 2014). Interestingly, DNA adduct formation by 3-NBA was not different in HCT116 *TP53(*+*/*+*)* and *TP53(*−*/*−*)* cells (Hockley et al. 2008), suggesting that NAD(P)H:quinone oxidoreductase (NQO1), which is the principal enzyme activating 3-NBA (compare Supporting Figure 1b) (Arlt et al. 2005; Stiborova et al. 2010), is not regulated by p53.

Transgenic and knockout mouse models have been used to study tumour suppressor function through phenotypic analysis of the whole organism and by examining a variety of primary cell types (Taneja et al. 2011). The opportunity to study multiple tissues is particularly useful for *Trp53* because p53 function is highly cell type specific (Donehower 2014; Kenzelmann Broz and Attardi 2010; Kucab et al. 2010; Lozano 2010). Much of the work carried out on the role of CYP enzymes in xenobiotic metabolism has been done in vitro (Nebert 2006; Nebert and Dalton 2006). However, extrapolation from in vitro data to in vivo pharmacokinetics requires additional factors to be considered such as route of administration, absorption, renal clearance and tissue-specific CYP expression (Nebert 2006; Nebert et al. 2013). For example, previous studies have revealed an apparent paradox, whereby hepatic CYP enzymes appear to be more important for detoxification of BaP in vivo, despite being involved in its metabolic activation in vitro (Arlt et al. 2008, 2012; Nebert et al. 2013).

To evaluate the impact of the cellular *Trp53* status on the metabolic activation of BaP and 3-NBA, we have compared metabolism and DNA adduct formation of BaP and 3-NBA in *Trp53(*+*/*+*)*, *Trp53(*+*/*–*)* and *Trp53(*−*/*−*)* mice. DNA adduct formation in vivo and in vitro was investigated by ^32^P-postlabelling analysis. Tissue-specific expression and activity of xenobiotic-metabolising enzymes (XMEs) involved in BaP and 3-NBA metabolism were compared with DNA adduct formation in the same tissue. Nucleotide excision repair (NER) capacity was assessed phenotypically in selected tissues using a modified comet assay. Urinary BaP metabolites and the Cyp-mediated formation of BaP metabolites ex vivo in hepatic microsomes were measured by high-performance liquid chromatography (HPLC).

## Materials and methods

### Carcinogens

Benzo[*a*]pyrene (BaP; CAS number 50-32-8; purity ≥96 %) was obtained from Sigma-Aldrich. 3-Nitrobenzanthrone (3-NBA; CAS number 17117-34-9) was prepared as previously reported (Arlt et al. 2002). (±)-*Anti*-benzo[*a*]pyrene-*trans*-7,8-dihydrodiol-9,10-epoxide (BPDE) was synthesised at the Biochemical Institute for Environmental Carcinogens, Prof. Dr. Gernot Grimmer-Foundation, Germany.

### Carcinogen treatment of *Trp53(*+*/*+*)*, *Trp53(*+*/*−*)* and *Trp53(*−*/*−*)* mice

*Trp53(*+*/*+*)*, *Trp53(*+*/*–*)* and *Trp53(*−*/*−*)* male C57BL/6 mice were generated as reported (Jacks et al. 1994). *Trp53(*+/−*)* and *Trp53(*−*/*−*)* mice carry a mutation which removes approximately 40 % of the coding capacity of *Trp53* and completely eliminates synthesis of p53 protein. More information about the strains can be found at (http://jaxmice.jax.org/strain/002101.html). All animal experiments were conducted in accordance with the law at the Leiden University Medical Center, Leiden, the Netherlands, after approval by the institutional ethics committee. Animals were kept under controlled specific pathogen-free conditions (23 °C, 40–50 % humidity) under a 12-h light–dark cycle. Food and water were available ad libitum. Genotyping of the animals was performed as described (Jacks et al. 1994) (see Supporting Figure 2). Groups of male *Trp53(*+*/*+*)*, *Trp53(*+*/*–*)* and *Trp53(*−*/*−*)* mice (3 months old; 25–30 g; *n* = 4/group) were treated with a single dose of 125 mg/kg body weight (bw) of BaP by intraperitoneal (i.p.) injection according to treatment protocols used previously to study BaP metabolism (Arlt et al. 2008, 2012). We chose i.p. injection as the administration route to achieve a high induction of hepatic CYP-mediated BaP metabolism. Similarly, groups (*n* = 4) of *Trp53(*+*/*+*)*, *Trp53(*+*/*–*)* and *Trp53(*−*/*−*)* mice were injected i.p. with a single dose of 2 mg/kg bw of 3-NBA according to a previous study investigating 3-NBA metabolism (Arlt et al. 2005). Based on dose-finding experiments in *Trp53(*+*/*+*)* mice using single i.p. injections of 1.25, 6.25 or 12.5 mg/kg bw of BPDE, groups (*n* = 4) of *Trp53(*+*/*+*)*, *Trp53(*+*/*–*)* and *Trp53(*−*/*−*)* mice were treated i.p. with 1.25 mg/kg bw of BPDE. Control mice (*n* = 4) received solvent (corn oil) only. Animals were killed 24 h after treatment, and their liver, lung, kidney, colon, small intestine, bladder, glandular stomach, forestomach and spleen were removed, snap-frozen in liquid nitrogen and stored at −80 °C until further analysis. Urine was collected for the preceding 24 h.

### Detection of DNA adducts by ^32^P-postlabelling

DNA was isolated from tissues by a standard phenol–chloroform extraction method. DNA adduct analysis was performed by thin-layer chromatography ^32^P-postlabelling analysis (Phillips and Arlt 2007, 2014). For DNA from BaP- and BPDE-treated animals, the nuclease P1 digestion enrichment method was used (Arlt et al. 2008), while for DNA from 3-NBA-treated animals, the butanol extraction method was employed (Arlt et al. 2002). DNA samples (4 μg) were digested with micrococcal nuclease (288 mU; Sigma) and calf spleen phosphodiesterase (1.2 mU; MP Biomedical) and then enriched and labelled as reported.

### Measurement of nucleotide excision repair (NER) capacity

The ability of NER-related enzymes present in isolated tissue extracts to detect and incise substrate DNA containing BPDE-DNA adducts was measured using a modified comet assay (Langie et al. 2006). Tissue protein extracts were prepared as described previously (Güngör et al. 2010), and protein concentrations were optimised for analysis of liver and kidney samples (0.2 mg/mL). The ex vivo repair incubation and electrophoresis were performed according to the published protocol (Langie et al. 2006). Dried slides stained with ethidium bromide (10 µg/mL) were viewed with a Zeiss Axioskop fluorescence microscope. Comets were scored using the Comet III system (Perceptive Instruments, UK). Fifty nucleoids were assessed per slide, and each sample was analysed in duplicate. All samples were measured blindly. Tail intensity (% tail DNA), defined as the percentage of DNA migrated from the head of the comet to the tail, was used to calculate repair capacity of the tissue extracts as reported previously (Langie et al. 2006).

### Preparation of microsomal and cytosolic samples

Microsomal and cytosolic fractions were isolated from the livers and lungs of *Trp53(*+*/*+*)*, *Trp53(*+*/*–*)* and *Trp53(*−*/*−*)* mice. Tissue frozen in liquid nitrogen was ground up in a Teflon container with a steel ball in a dismembrator to a frozen powder. This was transferred into a Potter-Elvehjem homogenizer and the Teflon receptacle rinsed with 1/15 M sodium phosphate buffer with 0.5 % potassium chloride pH 7.4. The powder was homogenised and transferred to a centrifuge tube and the potter rinsed with phosphate buffer. The homogenates were spun for 30 min at 18,000×*g*. Supernatant were transferred to an ultracentrifuge tube and spun at 100,000×*g* for 60 min at 4 °C. The resulting supernatants formed the cytosols, which was levered off the sediment gently, while the sediments (microsomes) were taken up in phosphate buffer. Protein concentration in the fractions was measured using bicinchoninic acid protein assay (Wiechelman et al. 1988) with bovine serum albumin and stored in small aliquots at −80 °C until analysis.

### Microsomal BaP-DNA adduct formation

Incubation mixtures in a final volume of 750 µL consisted of 50 mM potassium phosphate buffer (pH 7.4), 1 mM NADPH, 0.5 mg of microsomal protein, 0.5 mg calf thymus DNA and 0.1 mM BaP [dissolved in 7.5 µL dimethylsulfoxide (DMSO)]. The reaction was initiated by adding NADPH. Microsomal incubations were carried out at 37 °C for 90 min. Microsomal-mediated BaP-DNA adduct formation was linear up to 120 min as reported previously (Arlt et al. 2008). Control incubations were carried out (*i*) without microsomes, (*ii*) without NADPH, (*iii*) without DNA and (*iv*) without BaP. After incubation, DNA was isolated by a standard phenol/chloroform extraction method.

### Microsomal BaP metabolite formation

In a final volume of 500 µL, the incubation mixture contained 100 mM potassium phosphate buffer (pH 7.4), NADPH-generating system (1 mM NADP^+^, 10 mM d-glucose-6-phosphate, 1 μ/mL d-glucose-6-phosphate dehydrogenase), 0.5 mg microsomal protein and 50 µM BaP (dissolved in 5 µL DMSO). The reaction was initiated by adding 50 µL of the NADPH-generating system. Microsomal incubations were carried out at 37 °C for 20 min. Control incubations were carried out (1) without microsomes, (2) without NADPH-generating system and (3) without BaP. After incubation, 5 µL of 1 mM phenacetin in methanol was added as internal standard. The BaP mixtures were extracted with ethyl acetate (2 × 1 mL), the solvent was evaporated to dryness, and the residue was dissolved in 25 µL methanol for HPLC analysis.

### HPLC analysis of BaP metabolites

HPLC analysis was performed on a Nucleosil^®^ C18 reversed phase column (250 × 4 mm, 5 µm; Macherey Nagel, Germany), using a Dionex system consisting of a pump P580, a UV/VIS Detector UVD 170S/340S, an ASI-100 Automated Sample Injector, a termobox COLUMN OVEN LCO 101 and an In-Line Mobile Phase Degasser Degasys DG-1210 Dionex controlled with ChromeleonTM 6.11 build 490 software. HPLC conditions were as follows: 50 % acetonitrile in HPLC water (*v*/*v*), with a linear gradient to 85 % acetonitrile in 35 min, then an isocratic elution with 85 % acetonitrile for 5 min, a linear gradient from 85 % acetonitrile to 50 % acetonitrile in 5 min, followed by an isocratic elution of 50 % acetonitrile for 5 min. Detection was by UV at 254 nm. BaP metabolite peaks were collected and analysed by NMR and/or mass spectrometry as described (Stiborova et al. 2014). The structures of BaP metabolites analysed are given in Supplementary Figure 8. The metabolite peak areas were calculated relative to the peak area of the internal standard.

### BaP metabolites in urine

Urine samples (0.4–1.7 mL) collected from *Trp53(*+*/*+*)* and *Trp53(*−*/*−*)* mice treated with BaP were mixed with four volumes of methanol and centrifuged for 4 min at 1000 rpm, and the supernatants were then evaporated to dryness. The residues were dissolved in 100 µL of methanol and analysed by HPLC as described above. Urine samples for *Trp53(*+*/*–*)* mice were lost during analysis.

### Expression of Cyp1a1 and Nqo1 by Western blotting

Microsomal and cytosolic proteins were separated using NuPage 4–12 % Bis–Tris sodium-dodecyl sulphate (SDS)-polyacrylamide gels (Life Technologies) and Western-blotted as previously reported (Hockley et al. 2006). Chicken polyclonal antibody raised against recombinant rat CYP1A1 protein (Arlt et al. 2008) has been shown to recognise murine Cyp1a1. In microsomal samples, Cyp1a1 was probed with chicken anti-rat CYP1A1 at 1:5000, and peroxidase-conjugated goat anti-chicken (ab6877, Abcam, 1:10,000) was used as secondary antibody. Rat recombinant CYP1A1 and CYP1A2 (in Supersomes™, Gentest Corp.) were used as positive controls to identify protein bands in microsomal samples. In cytosolic samples, an affinity-purified rabbit antibody was used to detect Nqo1 (N5288, rabbit pAb, 1:10,000; Sigma) and peroxidase-conjugated goat anti-rabbit antibody (CST7076, Cell Signalling Technology, 1:10,000) was used as secondary antibody. Human recombinant NQO1 (Sigma) was used as positive control to identify the nqo1 band in cytosols. Gapdh was detected with mouse mAb #MAB374 (1:10,000; Millipore) and β-actin with mouse mAb ab6276 (1:10,000; Abcam) using peroxidase-conjugated goat anti-mouse as secondary antibody #170-5047 (1:5000; Bio-Rad). All proteins were visualised using the enhanced chemiluminescent SuperSignal West Pico detection reagent according to the manufacturer’s instruction (#34080; Thermo Scientific).

### Measurement of Cyp1a1 enzyme activity

Microsomal samples were characterised for Cyp1a activity using 7-ethoxyresorufin *O*-deethylation (EROD) activity (Mizerovska et al. 2011). Enzyme activity was determined by following the conversion of 7-ethoxyresorufin into resorufin using fluorescent measurement on a Synergy HT Plate Reader (Bio-TEK) using an excitation wavelength of 530 nm and an emission wavelength of 580 nm.

### Measurement of Nqo1 enzyme activity

Nqo1 enzyme activity in cytosolic samples was measured with menadione (2-methyl-1,4-naphthoquinone) as substrate as described previously (Mizerovska et al. 2011). Enzyme activity was determined by following the conversion of cytochrome *c* at 550 nm on a Synergy HT Plate Reader (Bio-TEK).

### Expression of p53 by Western blotting

For the preparation of whole hepatic protein, extracts of liver tissues (30 mg) of *Trp53(*+*/*+*)* mice were homogenised in 300 µL of Tissue Protein Extraction Reagent (T-PER™, Life Technologies) buffer supplemented with 1 % protease inhibitor (Halt™, Life Technologies). Samples were sonicated and centrifuged for 20 min at 13,000*g* (4 °C), and the supernatant was saved. The protein concentration was measured as described above. For Western blotting, 25 µg of protein was separated by SDS–polyacrylamide electrophoresis as described above. The following antibodies were used: anti-p53 (rabbit pAb, NCL-p53-CM5p, 1:5000; Leica Biosystems) and anti-Gadph (mouse mAb #MAB374, 1:10,000; Millipore). Membranes were washed and incubated with peroxidase-conjugated goat anti-rabbit or goat anti-mouse as secondary antibodies (#170-5046 and #170-5047, 1:5000; Bio-Rad).

## Results

### DNA adduct formation in vivo

In the majority of tissues, the BaP-DNA adduct pattern consisted of a single adduct spot (spot 1), previously identified as 10-(deoxyguanosin-*N*^2^-yl)-7,8,9-trihydroxy-7,8,9,10-tetrahydro-BaP (dG-*N*^2^-BPDE) (Supporting Figure 3a). For lung, colon and small intestine additional adduct spots were detected. In all three tissues, a minor adduct (spot 2) was detected that was previously suggested to be derived from reaction of 9-hydroxy-BaP-4,5-epoxide with guanine (Stiborova et al. 2014), while for colon and small intestine, an additional major adduct (spot 3) was found that has not yet been structurally identified. The same adduct profiles were observed in all three mouse lines. A scheme showing the formation of adducts 1 and 2 is given in Supporting Figure 6. No DNA adducts were detected in control animals (data not shown).

**Fig. 1:**
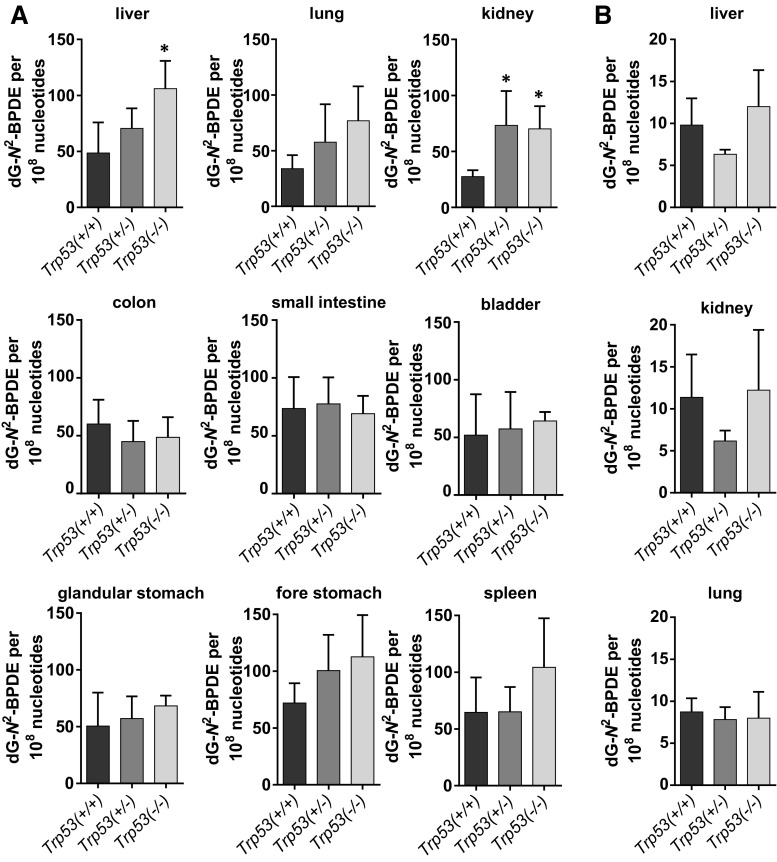
DNA adduct levels measured by ^32^P-postlabelling in various organs of *Trp53(*+*/*+*)*, *Trp53(*+*/*–*)* and *Trp53(*−*/*−*)* mice after exposure to BaP (**a**) or BPDE (**b**). Values are the mean ± SD (*n* = 4). Statistical analysis was performed by one-way ANOVA followed by Tukey post hoc test [*p < 0.05; different from *Trp53(*+*/*+*)* mice]

BaP-DNA adduct levels ranged from 25 to 100 adducts per 10^8^ nucleotides (Fig. 1a). Adduct levels were significantly higher (~ twofold) in livers of *Trp53(*−*/*−*)* compared to *Trp53(*+*/*+*)* mice (106 ± 25 *versus* 48 ± 27 adducts per 10^8^ nucleotides; *p* < 0.05), while adduct formation in kidney was significantly higher (~ twofold) in both *Trp53(*+*/*–*)* and *Trp53(*−*/*−*)* relative to *Trp53(*+*/*+*)* mice (73 ± 31 and 70 ± 20 *versus* 27 ± 6 adducts per 10^8^ nucleotides, respectively; *p* < 0.05). A similar trend was observed in lung, fore stomach and spleen, although the difference in adduct levels did not reach statistical significance. In contrast, adduct levels in liver, lung and kidney did not significantly change after treatment with BPDE in all mouse lines (Fig. 1b). In these tissues, the adduct pattern consisted of adduct spot 1 only (see Supporting Figure 3b). As BPDE does not require CYP-mediated metabolic activation to bind to DNA, these findings suggest that the differences in DNA adduct formation observed in liver and kidney with the parent compound BaP are the consequence of the different capacities of the *Trp53* mouse lines to metabolically activate BaP.

**Fig. 2:**
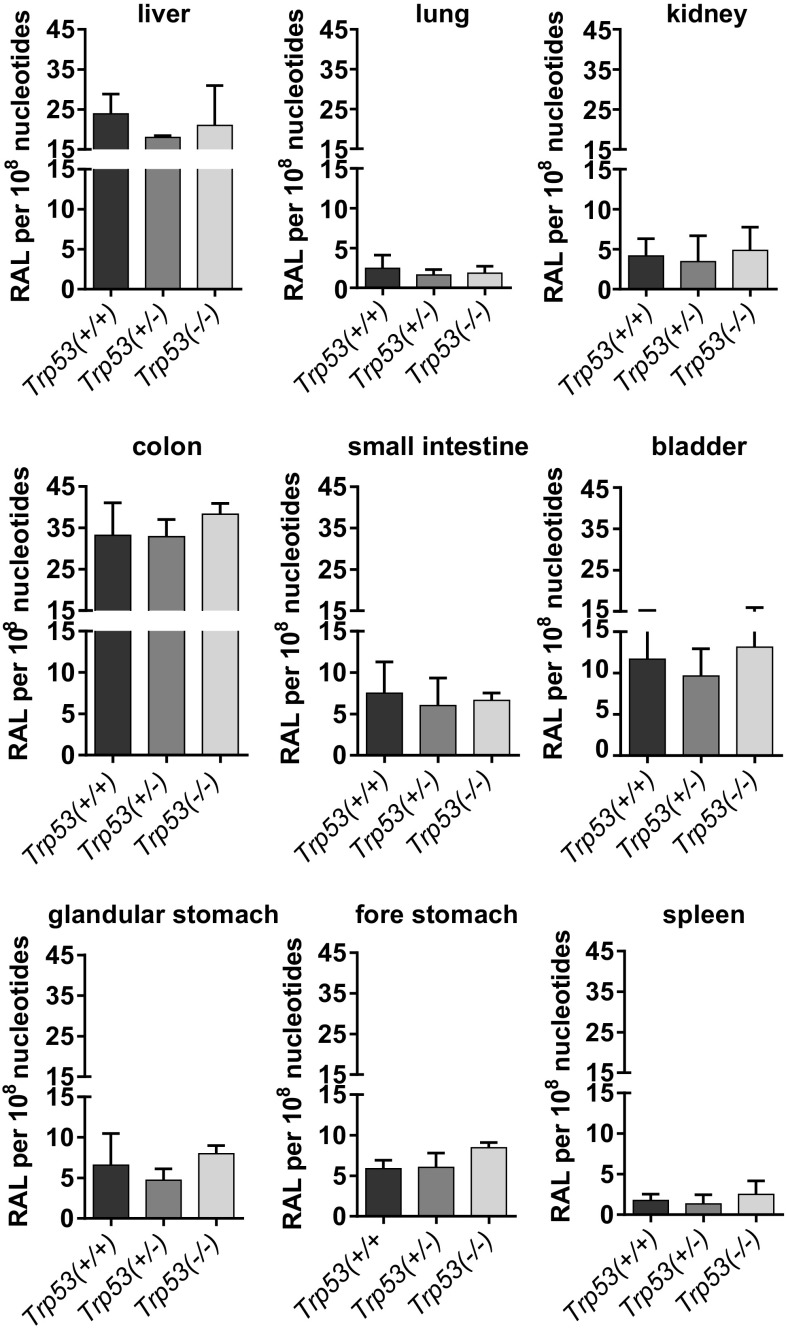
DNA adduct levels measured by ^32^P-postlabelling in various organs of *Trp53(*+*/*+*)*, *Trp53(*+*/*–*)* and *Trp53(*−*/*−*)* mice after exposure to 3-NBA. Values are the mean ± SD (*n* = 4). Statistical analysis was performed by one-way ANOVA followed by Tukey post hoc test; no significant differences were observed

The adduct pattern induced by 3-NBA consisted of up to four adduct spots (spots 1–4; Supporting Figure 4). These were characterised previously as 2-(2′-deoxyadenosin-*N*^6^-yl)-3-aminobenzanthrone (dA-*N*^6^-3-ABA; spot 1), 2-(2′-deoxyguanosin-*N*^2^-yl)-3-aminobenzanthrone (dG-*N*^2^-3-ABA; spot 3) and *N*-(2′-deoxyguanosin-8-yl)-3-aminobenzanthrone (dG-C8-*N*-3-ABA; spot 4), while spot 2 is an as-yet-uncharacterised deoxyadenosine adduct (Arlt et al. 2001, 2006). Adduct levels ranged from 2 to 40 adducts per 10^8^ nucleotides, but there were no significant differences between mouse lines in DNA adduct formation in any of the tissues investigated (Fig. 2). These results suggest that in contrast to BaP metabolism, *Trp53* status has no impact on 3-NBA metabolism in vivo, which is in accord with experiments on human cells in vitro (Hockley et al. 2008; Simoes et al. 2008).

### DNA repair capacity in liver and kidney

As p53-dependent pathways affecting global NER have been identified (Ford 2005; Sengupta and Harris 2005), we assessed whether mouse *Trp53* status influences NER activity. Tissue extracts from liver and kidney were examined for their ability to repair BPDE-induced DNA adducts using a modified comet assay (Langie et al. 2006). We found that in the liver, the repair capacity was ~50 % higher in *Trp53(*−*/*−*)* than in *Trp53(*+*/*+*)* mice, while no difference in the repair capacity between mouse lines was observed in kidney (Supporting Figure 12).

### DNA adduct formation of BaP ex vivo

**Fig. 3:**
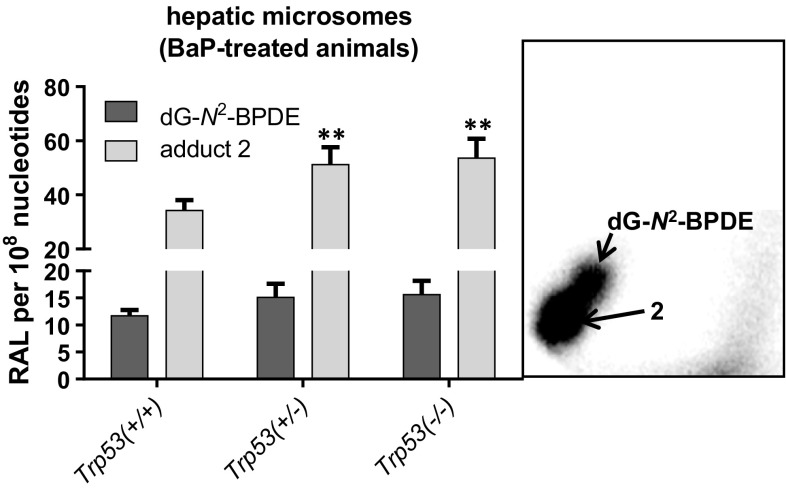
BaP-DNA adducts, measured by ^32^P-postlabelling, formed ex vivo by hepatic microsomes isolated from BaP-pretreated *Trp53(*+*/*+*)*, *Trp53(*+*/*–*)* and *Trp53(*−*/*−*)* mice. Values are the mean ± range (*n* = 4); duplicate incubations and each sample were determined by two independent post-labelled analyses. Statistical analysis was performed by one-way ANOVA followed by Tukey post hoc test [**p < 0.01; different from hepatic microsomes isolated from *Trp53(*+*/*+*)* mice]. *Insert* autoradiographic profiles of DNA adducts formed in hepatic microsomes isolated from *Trp53(*+*/*+*)* mice; the origins, at the *bottom left-hand* corners, were cut off before exposure

We investigated the ability of hepatic microsomes isolated from both control and BaP-treated animals to catalyse BaP-DNA adduct formation ex vivo (Fig. 3; Supporting Figure 5). While *Trp53* status had no influence on DNA adduct formation by BaP with hepatic microsomes isolated from untreated animals (Supporting Figure 5), BaP-induced adduct levels were significantly higher with microsomal samples from *Trp53(*+*/*–*)* and *Trp53(*−*/*−*)* relative to *Trp53(*+*/*+*)* mice pretreated with BaP (Fig. 3). The adduct pattern induced by BaP ex vivo consisted of adduct spots 1 and 2 (see inserts Fig. 3 and Supporting Figure 5). Interestingly, adduct levels of adduct 2 were higher than adduct 1, the dG-*N*^2^-BPDE adduct, and, in addition, adduct 2 was significantly higher in experiments with *Trp53(*+*/*–*)* and *Trp53(*−*/*−*)* microsomes than with *Trp53(*+*/*+*)* microsomes.

### BaP metabolite profile ex vivo

As our data suggested that *Trp53* status affects the NADPH-dependent metabolic activation of BaP in hepatic microsomes, metabolite profiles were determined by HPLC analysis. First, we investigated whether the hepatic microsomes isolated from BaP-treated mice contained residual BaP and/or its metabolites. No amounts of BaP and BaP metabolites were detectable in these microsomal fractions (Supporting Figure 7c–e). To determine the BaP metabolite profile, hepatic microsomes isolated from *Trp53(*+*/*+*)*, *Trp53(*+*/*–*)* and *Trp53(*−*/*−*)* were incubated with BaP and subsequently analysed by HPLC analysis. A representative HPLC chromatogram of BaP metabolites formed in ex vivo incubations containing microsomes, NADPH and BaP is shown in Supporting Figure 7a.

**Fig. 4:**
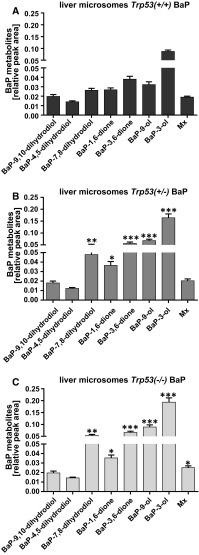
Formation of BaP metabolites by hepatic microsomes isolated from BaP-treated *Trp53(*+*/*+*)* (**a**), *Trp53(*+*/*–*)* (**b**) and *Trp53(*−*/*−*)* mice (**c**). Relative peak areas of BaP metabolites were measured by HPLC analysis at 254 nm. Values are the mean ± SD (*n* = 3). Statistical analysis was performed by one-way ANOVA followed by Tukey post hoc test [*p < 0.05, **p < 0.01, ***p < 0.005; different from BaP-treated *Trp53(*+*/*+*)* mice]. Structures of the BaP metabolites detected by HPLC are shown in Supplementary Figure 8. Mx, an unknown BaP metabolite

Hydroxylated BaP metabolites, BaP-dihydrodiols and BaP-diones were identified (see Supporting Figure 8). Previous studies have shown that many of these BaP metabolites are formed by CYP1A1 in combination with microsomal epoxide hydrolase (Bauer et al. 1995; Kim et al. 1998; Luch and Baird 2005). No BaP metabolites were detected in control incubations without microsomes, without NADPH-generating system or without BaP (Supporting Figure 7b, c). With hepatic microsomes isolated from untreated mice, *Trp53* status had no influence on the BaP metabolite profile and the amounts of BaP metabolites formed (Supporting Figure 9). However, pretreating mice with BaP led to a significant increase in BaP metabolite formation ex vivo (Fig. 4). Moreover, amounts of BaP-7,8-dihydrodiol, BaP-1,6-dione, BaP-3,6-dione, BaP-9-ol and BaP-3-ol were significantly higher in microsomes of pretreated *Trp53(*+*/*–*)* and *Trp53(*−*/*−*)* mice compared with pretreated *Trp53(*+*/*+*)* mice. These findings correlated with ex vivo BaP-DNA adduct formation using the same hepatic microsomes (compare Fig. 3).

### Urine analysis of *Trp53*(+*/*+) and *Trp53(*−*/*−*)* mice treated with BaP

A representative HPLC chromatogram of urinary BaP metabolites is shown in Supporting Figure 13a. The amounts of BaP-4,5-dihydrodiol, BaP-7,8-dihydrodiol, BaP-3,6-dione and BaP-3-ol were significantly lower in urine of *Trp53(*−*/*−*)* compared to *Trp53(*+*/*+*)* mice (Supporting Figure 13b), although most of the differences were small (~15–20 % reduction). BaP metabolites can be conjugated to glucuronides and sulphates (Luch and Baird 2005), which are excreted in the urine and faeces, but only unconjugated BaP metabolites (see Supporting Figure 8) were determined in the present work.

### Influence of *Trp53* status on xenobiotic-metabolising enzymes

We next studied the expression and activity of enzymes metabolising BaP. Metabolic activation of BaP is catalysed mainly by CYP1A1, but also CYP1B1 (Supporting Figure 1a), which leads via BPDE to the formation of dG-*N*^2^-BPDE, the major BaP-derived DNA adduct detected in vivo. For these investigations, we selected liver, as *Trp53* status impacted on hepatic BaP-DNA adduct formation (see Fig. 1a), and also lung, because it is the main target organ of 3-NBA genotoxicity (Arlt 2005).

**Fig. 5:**
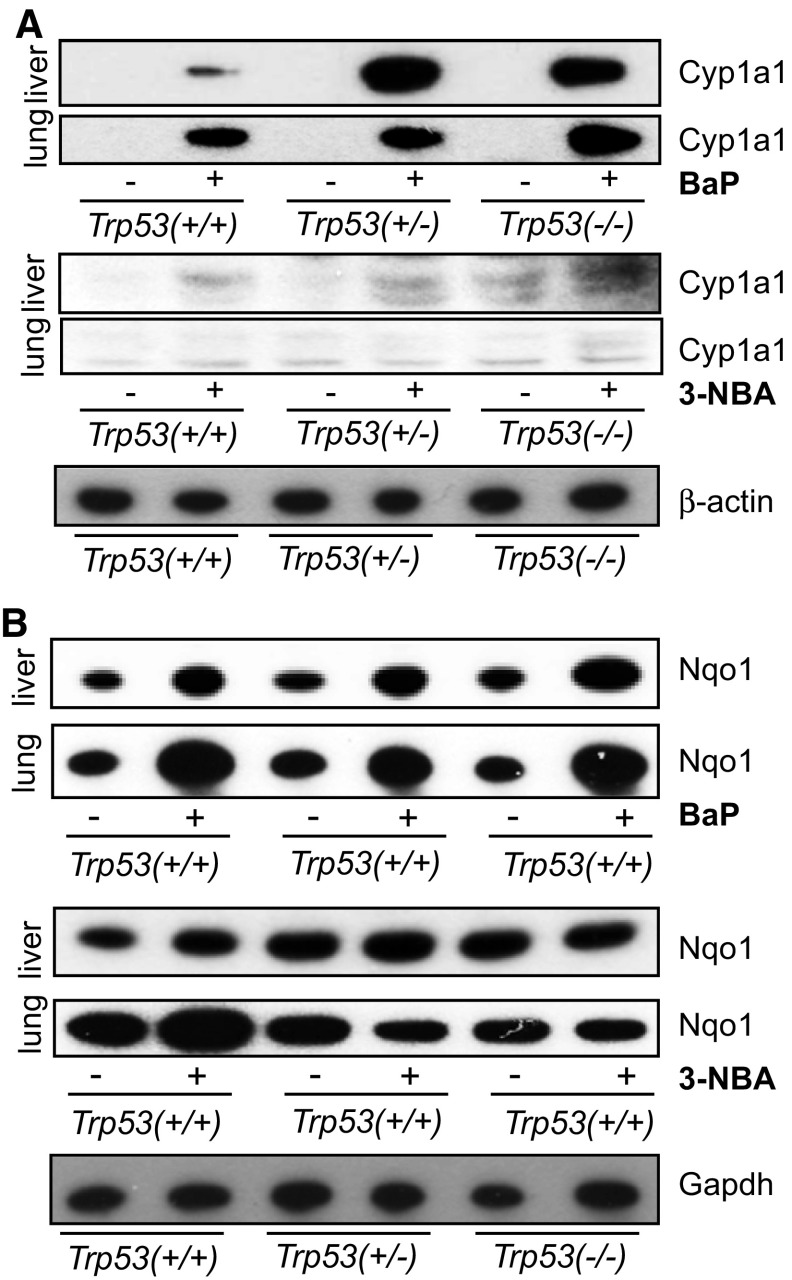
Western blot analysis of Cyp1a1 (**a**) and Nqo1 protein expression (**b**) in hepatic and pulmonary cytosols isolated from *Trp53(*+*/*+*)*, *Trp53(*+*/*–*)* and *Trp53(*−*/*−*)* mice exposed to BaP or 3-NBA. Representative images of the Western blotting are shown, and at least duplicate analysis was performed from independent experiments. β-Actin protein expression was used as a loading control of the microsomal fractions and Gadph for the cytosolic fractions, and a representative blot is shown

**Fig. 6:**
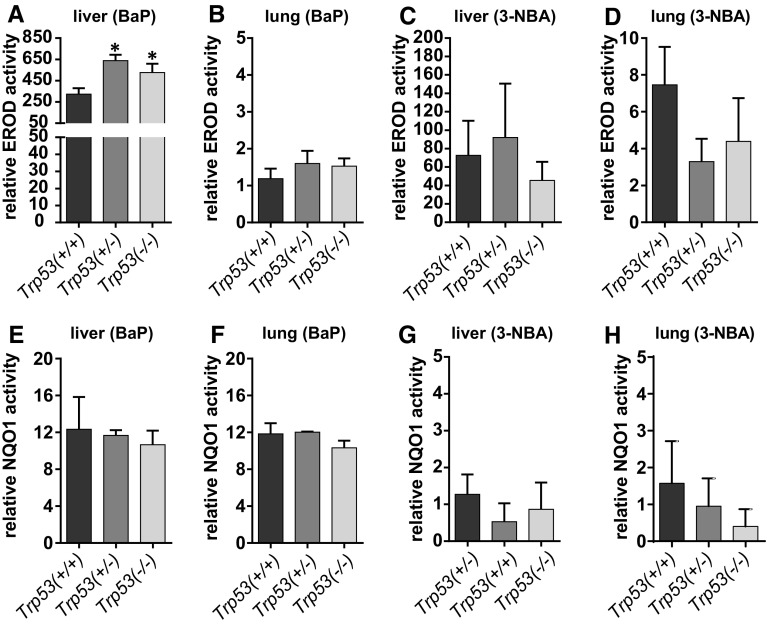
EROD activity (**a**–**d**) in hepatic (**a**, **c**) and pulmonary microsomes (**b**, **d**) isolated from *Trp53(*+*/*+*)*, *Trp53(*+*/*–*)* and *Trp53(*−*/*−*)* mice exposed to BaP or 3-NBA. Nqo1 enzyme activity (**e**–**h**) was determined in hepatic (**e** and **g**) and pulmonary cytosols (**f** and **h**) isolated from *Trp53(*+*/*+*)*, *Trp53(*+*/*–*)* and *Trp53(*−*/*−*)* mice exposed to BaP or 3-NBA. Values are the mean ± SD (*n* = 4). Statistical analysis was performed by one-way ANOVA followed by Tukey post hoc test [*p < 0.05; different from *Trp53(*+*/*+*)* mice]

Treatment of mice with BaP led to a large induction of Cyp1a1 protein levels in liver (Fig. 5a). However, induction was much greater in hepatic microsomes of BaP-treated *Trp53(*+*/*–*)* and *Trp53(*−*/*−*)* mice than of *Trp53(*+*/*+*)* mice, which correlates with the levels of BaP-DNA adducts in the livers of these animals. In this context, it may be noteworthy that no induction of p53 protein levels was observed after BaP treatment in the livers of *Trp53(*+*/*+*)* mice (Supporting Figure 10). The increase in Cyp1a1 protein levels was associated with a significant increase in EROD activity (up to ~ twofold; *p* < 0.05), a measure of CYP1A enzyme activity, in BaP-treated *Trp53(*+*/*–*)* and *Trp53(*−*/*−*)* compared with *Trp53(*+*/*+*)* mice (Fig. 6a). In contrast, *Trp53* status had no effect on EROD activity in hepatic microsomes isolated from control (untreated) mice (see Supporting Figure 11a), which was in line with no changes being observed in ex vivo BaP-DNA adduct formation (compare Supporting Figure 5) and BaP metabolite formation ex vivo using the same microsomes (compare Supporting Figure 9). Cyp1a1 protein levels were increased in pulmonary microsomes isolated from BaP-treated *Trp53(*−*/*−*)* compared to *Trp53(*+*/*+*)* (Fig. 5a), but no significant change in EROD activity was observed (Fig. 6b). Lung microsomes isolated from control (untreated) mice showed variability in EROD activity between preparations and/or experiments (compare Supporting Figure 11b, d) which may also be linked to low basal activity in these microsomes. No changes for Cyp1a1 protein levels, which were close to background levels (Fig. 5a), or enzyme activity (Fig. 6c, d) were observed in hepatic and pulmonary microsomes isolated from mice treated with 3-NBA between *Trp53* genotypes.

The principal enzyme that activates 3-NBA by nitroreduction is NQO1 (Supporting Figure 1b) (Arlt et al. 2005; Stiborova et al. 2010). The resulting *N*-hydroxy-3-aminobenzanthrone (*N*–OH-3-ABA) can spontaneously form reactive nitrenium ions capable of forming DNA adducts. Alternatively, *N*–OH-3-ABA can be further activated by *N*-acetyltransferases or sulfotransferases, leading to the formation of the same reactive nitrenium ions (Arlt 2005). It has been shown that in vivo cytosolic nitroreduction of 3-NBA is more important than nitroreduction by microsomal NAD(P)H:cytochrome P450 oxidoreductase (Arlt et al. 2003, 2005). As we did not observe any differences in DNA adduct formation with 3-NBA, we measured the expression and activity of Nqo1 in hepatic and pulmonary cytosols. In addition, as BaP-derivatives can also be metabolised by NQO1 (Luch and Baird 2005), we also determined expression and activity of Nqo1 in mice exposed to BaP.

All cytosolic samples contained Nqo1 (Fig. 5b). In both liver and lung, BaP treatment led to an induction of Nqo1 (Fig. 5b) that was independent of the *Trp53* genotype of the animals. These findings were in line with increased Nqo1 enzyme activity in these animals after BaP treatment (compare Supporting Figure 11e, f); however, *Trp53* status had no effect on Nqo1 enzyme activity in either organ, with or without BaP pretreatment of the animals. In hepatic cytosols, Nqo1 protein levels were unchanged in 3-NBA-treated mice (Fig. 5b). Nqo1 protein levels were increased in the lungs of 3-NBA-treated *Trp53(*+*/*+*)* mice, which is in concordance with previous studies showing that 3-NBA exposure can induce NQO1 (Stiborova et al. 2006, 2008), but not in 3-NBA-treated *Trp53(*+*/*–*)* and *Trp53(*−*/*−*)* mice. Exposure to 3-NBA and *Trp53* genotype had no impact on Nqo1 enzyme activity in hepatic and pulmonary cytosols (Fig. 6g, h; Supporting Figure 11g, h); however, strong variability in Nqo1 activity was seen in lung cytosols isolated from 3-NBA-treated *Trp53(*+*/*+*)* mice (Supporting Figure 11h). Collectively, these results suggest that p53 expression has no impact on Nqo1-mediated bioactivation of 3-NBA, which correlates with the lack of influence of *Trp53* genotype on 3-NBA-DNA adduct formation in vivo (Fig. 2).

## Discussion

We used *Trp53(*+*/*+*)*, *Trp53(*+*/*–*)* and *Trp53(*−*/*−*)* mice to investigate the effect of p53 on BaP metabolism and DNA adduct formation induced by BaP in vivo. The BaP dose used in this study, 125 mg/kg bw, has been shown to be carcinogenic and able to induce mutagenicity in multiple organs after repeated administration (Hakura et al. 1998). We found that BaP-induced DNA adduct formation in liver and kidney was significantly higher in *Trp53(*−*/*−*)* mice than in *Trp53(*+*/*+*)* mice after acute BaP treatment. Similar trends in DNA adduct formation were seen in other tissues (e.g. lung), although the difference in adduct formation did not reach statistical significance. This is in contrast to another study examining the effect of pentachlorophenol on DNA adduct formation in *Trp53(*+*/*+*)* and *Trp53(*−*/*−*)* mice exposed to BaP (Ress et al. 2002). In that study, no influence of *Trp53* status on BaP-DNA adduct formation was evident in the tissues investigated, namely liver and lung. The discrepancy between the two studies could be attributable to the different dosing regimens (i.e. a higher BaP dose was used in our study), but otherwise remains unexplained at present. CYP1A1 is considered to be one of the key enzymes responsible for the metabolic activation of BaP in organisms (Luch and Baird 2005). Our data showed that higher BaP-DNA adduct levels in the livers of *Trp53(*−*/*−*)* mice relative to *Trp53(*+*/*+*)* mice correlated with higher hepatic Cyp1a protein levels and increased Cyp1a enzyme activity in these animals.

The amounts of BaP metabolites and BaP-DNA adduct levels formed in incubations using hepatic microsomes isolated from BaP-pretreated *Trp53(*−*/*−*)* and *Trp53(*+*/*–*)* mice were higher than when using those from BaP-pretreated *Trp53(*+*/*+*)* mice. This indicates again that Cyp activity was induced in *Trp53(*−*/*−*)* mice which correlated with higher BaP-DNA adduct formation in this mouse line. However, studying BaP-DNA adduct formation ex vivo, we found that adduct 2, but not formation of dG-*N*^2^-BPDE, was higher in experiments with *Trp53(*+*/*–*)* and *Trp53(*−*/*−*)* microsomes than with *Trp53(*+*/*+*)* microsomes, whereas dG-*N*^2^-BPDE levels were twice as high in livers of *Trp53(*−*/*−*)* mice relative to *Trp53(*+*/*+*)* mice in vivo. The presence of both adducts in the microsomal incubation ex vivo can be explained by different metabolic pathways, leading to the formation of the precursors of each adduct (see Supporting Figure 6). The levels of adduct 2 correlated well with Cyp1a enzyme activity in ex vivo incubations with hepatic microsomes because they are solely dictated by the Cyp1a-mediated formation of 9-hydroxy-BaP-4,5-epoxide (Stiborova et al. 2014). In contrast, formation of dG-*N*^2^-BPDE strongly depends on the activity of microsomal epoxide hydrolase (mEH) which catalyses the hydration of BaP-7,8-epoxide to BaP-7,8-dihydrodiol which is the precursor of BPDE (see Supporting Figure 6). The formation of BaP-7,8-dihydrodiol in vivo should be more effective than ex vivo because, in addition to mEH present in hepatic microsomes used in the ex vivo incubations, other EH isoenzymes can catalyse the hydration reaction in vivo. Nevertheless, our ex vivo incubations demonstrate that Cyp-mediated BaP-DNA adduct formation is dependent on *Trp53* status.

Studies investigating xenobiotic metabolism and/or carcinogen-DNA adduct formation in transgenic mice with altered *Trp53* status are sparse and have mainly used *Trp53(*+*/*–*)* mice (Ariyoshi et al. 2001; Carmichael et al. 2001; Mori et al. 2001; Sanders et al. 2001). In one study, DNA adduct levels induced by diethylstilbestrol (DES) were ~twofold higher in *Trp53(*+*/*–*)* mice than in *Trp53(*+*/*+*)* mice (Carmichael et al. 2001). Small differences in the protein expression of several Cyp enzymes including Cyp1a1/2, Cyp1b1, Cyp2b9 and Cyp3a11 were observed, and it was concluded that these differences in the expression of XMEs between *Trp53(*+*/*–*)* and *Trp53(*+*/*+*)* mice could have contributed to the higher DES-induced DNA adduct levels seen in *Trp53(*+*/*–*)* mice. In this context, it is noteworthy that we also found higher DNA adduct levels in *Trp53(*+*/*–*)* mice relative to *Trp53(*+*/*+*)* mice after BaP exposure. Another study found that hepatic Cyp1a protein induction was higher in female *Trp53(*+*/*–*)* mice than in *Trp53(*+*/*+*)* mice after treatment with another PAH, 3-methylcholanthrene (Ariyoshi et al. 2001). This finding is in line with our results, showing that Cyp1a protein levels and Cyp1a enzyme activity are increased in the livers of *Trp53(*+*/*–*)* animals after BaP exposure. Other studies found no difference in the Cyp-mediated metabolism of *N*-butyl-*N*-(4-hydroxy-butyl)nitrosamine, benzene, ethoxyquin or methacrylonitrile between *Trp53(*+*/*+*)* and *Trp53(*+*/*–*)* mice (Mori et al. 2001; Sanders et al. 2001).

Haploinsufficiency in *TP53* has been shown to promote tumour development (Berger and Pandolfi 2011) as reduction in p53 gene dosage can impact on a cell’s ability to respond to DNA damage (Berger et al. 2011). As such, survival of *Trp53(*+*/*–*)* mice shows an intermediate survival to that of *Trp53(*−*/*−*)* and *Trp53(*+*/*+*)* mice, and tumours that develop in *Trp53(*+*/*–*)* mice do not always display loss of the remaining wild-type allele (Berger et al. 2011). Interestingly, in kidney, *Trp53(*+*/*–*)* mice behaved like *Trp53(*−*/*−*),* showing higher BaP-DNA adduct levels relative to *Trp53(*+*/*+*)* mice, while adduct formation in the livers of *Trp53(*+*/*–*)* and *Trp53(*+*/*+*)* mice was similar but lower compared to *Trp53(*−*/*−*)* mice. However, both hepatic Cyp1a protein levels and Cyp1a enzyme activity were higher in BaP-treated *Trp53(*+*/*–*)* and *Trp53(*−*/*−*)* mice than in *Trp53(*+*/*+*)* mice, suggesting that in *Trp53(*+*/*–*)* animals, other factors besides Cyp1a expression may influence BaP-DNA adduct formation in the liver.

NER is considered to be the main DNA repair pathway for bulky DNA adducts (Kucab et al. 2015), and p53-dependent pathways affecting global genomic NER have been identified (Ford 2005; Sengupta and Harris 2005). A modified comet assay measuring the tissue-specific NER capacity in untreated (control) animals showed no impact of *Trp53* status in kidney but an significant increase in NER capacity in *Trp53(*−*/*−*)* mice relative to *Trp53(*+*/*+*)* mice in liver. Although it is possible that BaP treatment may impact on NER capacity, a recent in vivo study has shown no transcriptomic responses related to NER genes in mice exposed to BaP (van Kesteren et al. 2013). Therefore, our results suggest that tissue-specific NER capacity did not contribute to the higher BaP-DNA adduct levels observed in *Trp53(*−*/*−*)* mice than in *Trp53(*+*/*+*)* mice, in both liver and kidney. This conclusion was strengthened by the fact that DNA adduct formation in livers and kidneys of *Trp53(*+*/*+*)*, *Trp53(*+*/*–*)* and *Trp53(*−*/*−*)* mice exposed to the corresponding reactive metabolite of BaP, BPDE, resulted in similar adduct levels in all mouse lines. As treatment with BPDE bypasses the need for metabolic activation, these results again indicated that the level of p53 expression impacts on the metabolic activation of the parent compound, BaP, but not on DNA repair.

Previous studies conducted in a panel of isogenic human cells differing only with respect to their endogenous *TP53* status showed that complete loss of p53 function (i.e. *TP53(*−*/*−*)* cells) resulted in considerably lower BaP-DNA adduct levels compared to *TP53(*+*/*+*)* cells after BaP exposure (Hockley et al. 2008; Wohak et al. 2014). CYP1A1 protein expression was induced to a much greater extent in *TP53(*+*/*+*)* cells than in *TP53(*+*/*–*)* and *TP53(*−*/*−*)* cells. There were also significantly lower levels of BaP metabolites in the culture medium of *TP53(*+*/*–*)* and *TP53(*−*/*−*)* cells correlating with lower BaP-DNA adduct formation in these cell lines. It was also shown that exposure to BPDE resulted in similar adduct levels in all cell lines and that NER capacity did not contribute to the observed differences in BaP-DNA adduct formation as the repair capacity was the same in all cell lines (Wohak et al. 2014). Collectively, these results demonstrate the role of p53 in the CYP1A1-mediated metabolism of BaP in human cells.

However, the impact of p53 function on BaP metabolism is different in vitro and in vivo. Whereas loss of p53 function results in a decrease in BaP-DNA adduct formation in vitro, loss of p53 function in vivo leads to an increase in BaP-DNA adduct formation. Other studies (Arlt et al. 2008; Nebert et al. 2013) have revealed a paradox, whereby CYP enzymes (particularly CYP1A1) appear to be more important for detoxification of BaP in vivo, despite being involved in its metabolic activation in vitro, demonstrating that XMEs can have different effects on carcinogen metabolism in vitro and in vivo. Nevertheless, the mechanism involved in the altered expression and activity of Cyp1a enzymes by p53 function in mice is presently unclear. It may not always be possible to extrapolate from in vitro data to in vivo pharmacokinetics as additional factors need to be considered such as route of administration, absorption, renal clearance and tissue-specific CYP expression (Arlt et al. 2008). Additionally, it will be necessary to examine whether there are species-dependent differences between cultured human and mouse cells in the p53-dependent BaP metabolism in vitro.

Many studies have shown that BaP can induce CYP1A1 through binding to the aryl hydrocarbon receptor (Ahr), a transcription factor that also regulates the expression of a number of phase I and phase II XME genes (Shimizu et al. 2000; Wang et al. 2011). However, when BaP was administered i.p. to *Ahr(*−*/*−*)* mice, total BaP-DNA adduct levels were similar to those in *Ahr(*+*/*+*)* mice, although the formation of individual adduct spots determined by ^32^P-postlabelling varied (Kondraganti et al. 2003). In contrast, in other studies, BaP-induced adduct levels in the livers of *Ahr(*−*/*−*)* mice were significantly higher than those in *Ahr(*+*/*+*)* mice after oral administration (Sagredo et al. 2006, 2009). Thus, these studies provide evidence of a mechanism of BaP biotransformation that is Ahr independent. Whether this mechanism involves p53 function in vivo is currently untested and would require the creation of a *Ahr(*−*/*−*)*/*Trp53(*−*/*−*)* double-knockout mouse line.

We recently found that BaP-induced CYP1A1 expression in human cells can be regulated through p53 binding to a p53 response element (p53RE) in the regulatory region of *CYP1A1*, thereby enhancing its transcription and thus explaining the role of p53 function in BaP metabolism in vitro as described above (Wohak et al. 2014). Others have shown that p53 induces the activity of CYP3A4 in human cells via its binding to p53REs and the subsequent transcriptional enhancement of *CYP3A4* (Goldstein et al. 2013). However, the induction of Cyps like Cyp1a1 via p53 binding to a p53RE in the *Cyp1a1* promoter region fails to explain the impact of p53 on BaP metabolism in vivo (present study), as Cyp1a protein levels and Cyp1a enzyme activity were actually higher in *Trp53(*−*/*−*)* mice which lack p53 function suggesting that the mechanism is different in vitro and in vivo, at least in the rodent model.

It remains to be seen whether this observation is specific to BaP or a more general phenomenon. However, bioactivation and DNA adduct formation of 3-NBA in vivo were not influenced by *Trp53* status (present study) which is in concordance with previous findings that 3-NBA induces similar levels of DNA adducts in human *TP53(*+*/*+*)* and *TP53(*−*/*−*)* cell lines (Hockley et al. 2008; Simoes et al. 2008). This observation indicates that the cellular impact of p53 on carcinogen metabolism depends on the agent studied and/or that only certain XMEs depend on p53 function. The most efficient enzyme to activate 3-NBA to DNA adducts is NQO1 (Arlt et al. 2005; Stiborova et al. 2010), and thus, NQO1 seems to be an enzyme not affected by p53 expression. This is in line with the fact that overall Nqo1 protein levels and Nqo1 enzyme activity were not significantly different in the *Trp53(*+*/*+*)*, *Trp53(*+*/*–*)* and *Trp53(*−*/*−*)* mouse lines, after both BaP and 3-NBA treatment. Future studies will be required to address this question, thereby testing other environmental and dietary carcinogens both in vitro and in vivo.

In summary, we found that murine Cyp-mediated bioactivation of BaP in vivo is influenced by p53 function, thereby providing new fundamental insights into PAH-induced carcinogenesis. These results indicate that gene–environment interactions need to be taken into account with regard to xenobiotic metabolism. Our results are in line with studies demonstrating an emerging role for p53 in carcinogen metabolism in human cells in vitro. Whereas in human cells, BaP-induced CYP1A1 expression is regulated through p53 binding to a p53RE in the *CYP1A1* regulatory region, thereby enhancing its transcription, the mechanism involved in the altered expression and activity of the Cyp1a1 enzyme by p53 in vivo in mice has yet to be identified. Future investigations will need to assess whether the metabolic activation of other environmental carcinogens depends on p53 in vivo and where the differences lie between murine and human p53.

## Electronic supplementary material

Supplementary material 1 (PDF 756 kb)
