# Supplementary material for: The impact of p53 on DNA damage and metabolic activation of the environmental carcinogen benzo[a]pyrene: effects in Trp53(+/+), Trp53(+/–) and Trp53(−/−) mice
Source: Arch Toxicol. 2015 May 21;90:839–51. doi: 10.1007/s00204-015-1531-8 (PMC4785204; doi:10.1007/s00204-015-1531-8)
Supplement: Supplementary file 1 — Supplementary material 1 (PDF 756 kb) [file 204_2015_1531_MOESM1_ESM.pdf]

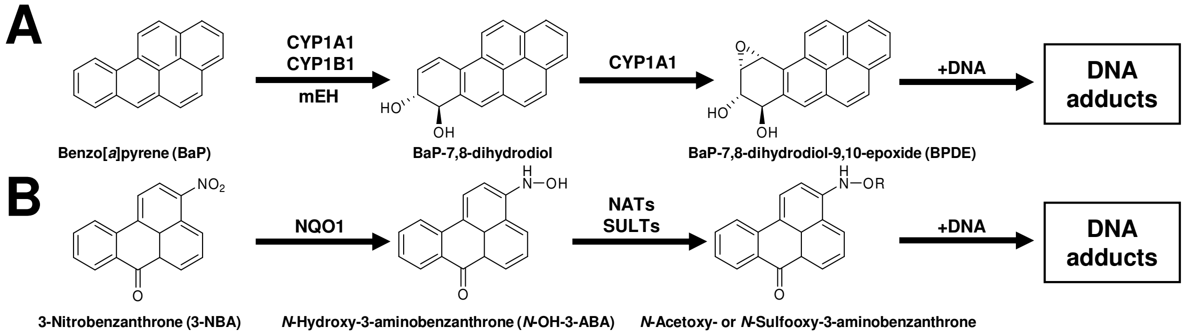

Supporting Figure 1

Main metabolic pathways in the bioactivation and DNA adduct formation *in vivo* of BaP (A) and 3-NBA (B). R = –C(O)CH_3; R = –SO_3H. See text for details. CYP, cytochrome P450; mEH, microsomal epoxide hydrolase; NQO1, NAD(P)H:quinone oxidoreductase; NAT, *N*-Acetyltransferase; SULT, sulfotransferase.

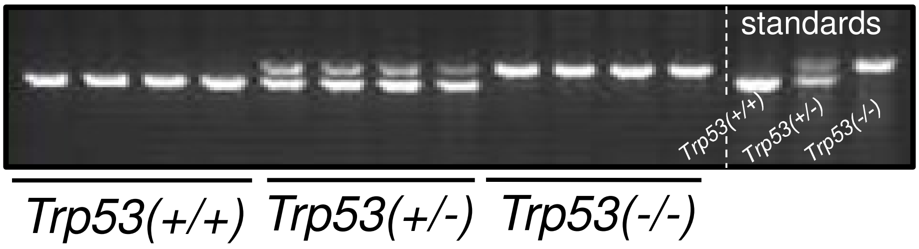

Supporting Figure 2

Genotyping example for the mouse *Trp53* allele in liver DNA isolated from *Trp53(+/+)*, *Trp53(+/-)* and *Trp53(-/-)* mice exposed to BaP. DNA was subjected to allele-specific primers and resolved on a 3% agarose gel containing ethidium bromide as described (Jacks et al. 1994).

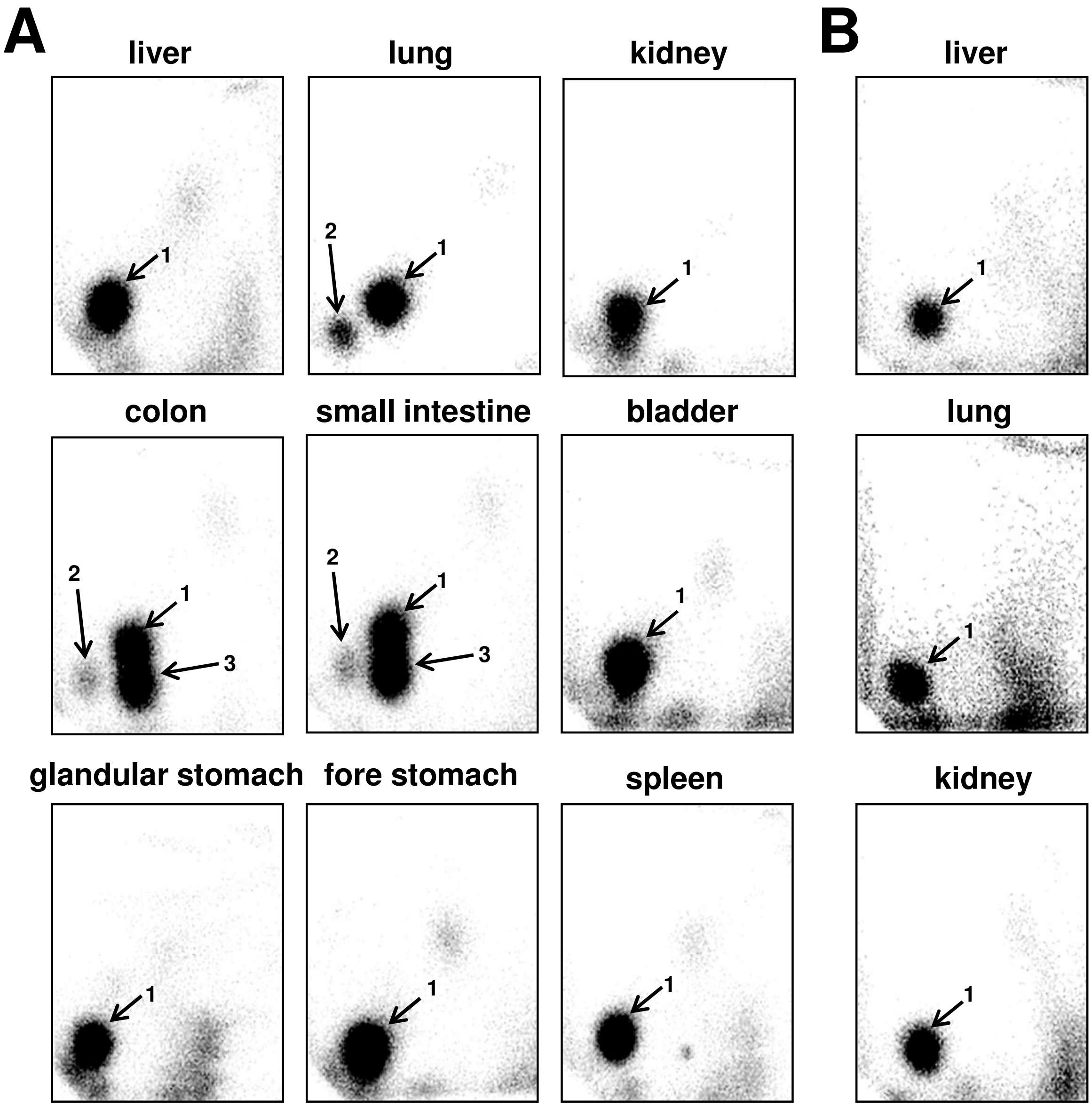

Supporting Figure 3

Autoradiographic profiles of DNA adducts, measured by ^{32}P-postlabelling, in various tissues of *Trp53(+/+)* mice exposed to BaP (A) or BPDE (B). The adduct profiles shown are representative for the same organs in *Trp53(+/-)* and *Trp53(-/-)* mice. Solvent conditions for the separation of BaP-derived DNA adducts were as follows: D1, 1.0 M sodium phosphate, pH 6.0; D3, 3.5 M lithium-formate, 8.5 M urea, pH 3.5; D4, 0.8 M lithium chloride, 0.5 M Tris, 8.5 M urea, pH 8.0. The origins, at the bottom left-hand corners, were cut off before exposure. Spot 1, 10-(deoxyguanosin-N^2-yl)-7,8,9-trihydroxy-7,8,9,10-tetrahydro-BaP (dG-N^2-BPDE); Spot 2, probable guanine adduct derived from reaction with 9-hydroxy-BaP-4,5-epoxide; Spot 3, uncharacterised BaP-derived DNA adducts. For the pathways of BaP-DNA adduct formation see Supporting Figure 6.

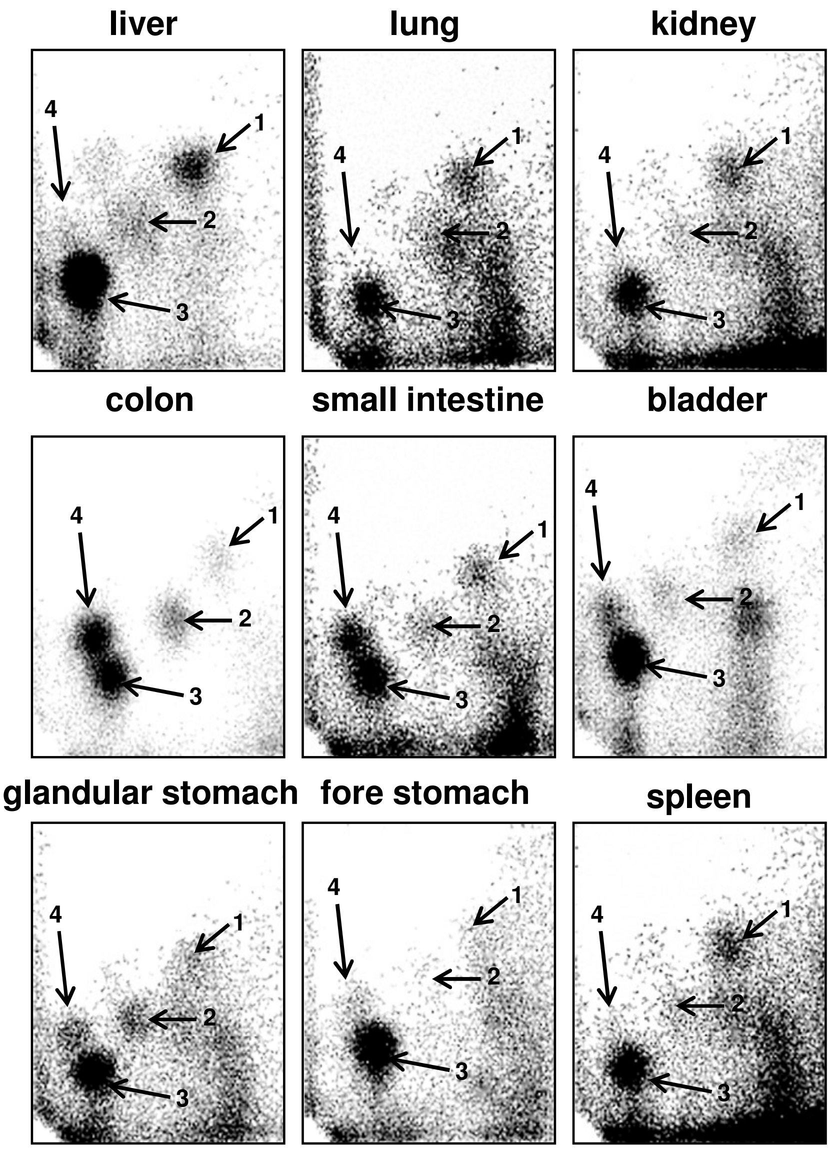

Supporting Figure 4

Autoradiographic profiles of DNA adducts, measured by ^{32}P-postlabelling, in various tissues of *Trp53(+/+)* mice exposed to 3-NBA. The adduct profiles shown are representative for the same organs in *Trp53(+/-)* and *Trp53(-/-)* mice. 3-NBA-derived DNA adducts were separated using the following solvent conditions: D1, 1.0 M sodium phosphate, pH 6.0; D3, 4.0 M lithium-formate, 7.0 M urea, pH 3.5; D4, 0.8 M lithium chloride, 0.5 M Tris, 8.5 M urea, pH 8.0. The origins, at the bottom left-hand corners, were cut off before exposure. Spot 1, 2-(2'-deoxyadenosine-N^6-yl)-3-aminobenzanthrone (dA-N^6-3-ABA); Spot 2, as-yet unidentified adenine adduct derived from nitroreduction; Spot 3, *N*-(2'-deoxyguanosine-N^2-yl)-3-aminobenzanthrone (dG-N^2-3-ABA); Spot 4, *N*-(2'-deoxyguanosin-8-yl)-3-aminobenzanthrone (dG-C8-*N*-3-ABA).

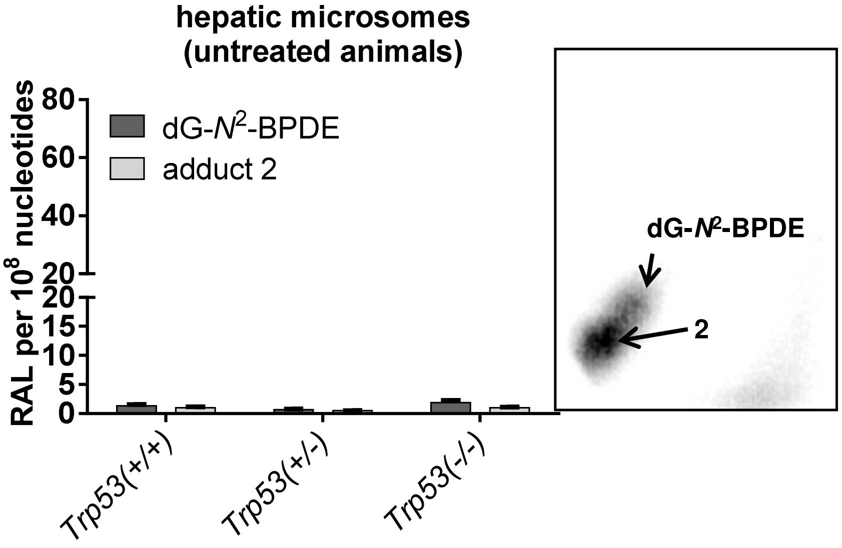

Supporting Figure 5

BaP-DNA adducts, measured by ^{32}P-postlabelling, formed *ex vivo* by hepatic microsomes isolated from control (untreated) *Trp53(+/+)*, *Trp53(+/-)* and *Trp53(-/-)* mice. Values are the mean ± range (n = 4); duplicate incubations and each sample was determined by two independent post-labelled analyses. Statistical analysis was performed by one-way ANOVA followed by Tukey post-hoc test; no significant differences were observed. Inserts: Autoradiographic profiles of DNA adducts formed in hepatic microsomes isolated from *Trp53(+/+)* mice; the origins, at the bottom left-hand corners, were cut off before exposure.

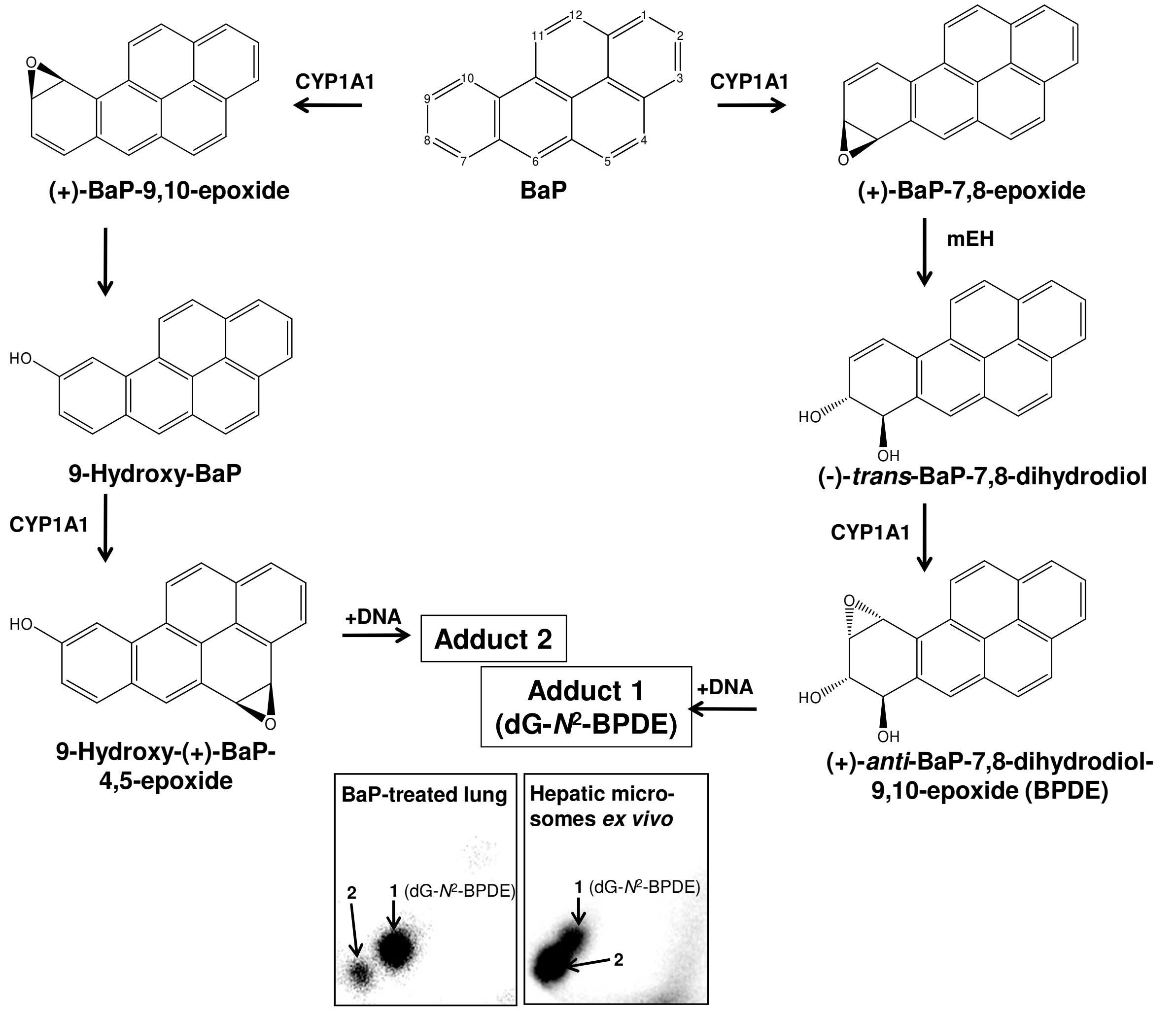

Supporting Figure 6

Pathways of biotransformation and DNA adduct formation of BaP catalysed by CYP1A1 and microsomal epoxide hydrolase (mEH) [adapted from (Stiborova et al. 2014)]. The typical three-step activation process with oxidation by CYP1A1 followed by hydrolysis by mEH leads to the ultimately reactive species BPDE which leads to the generation of the dG-N^2-BPDE adduct. The two-step activation process by CYP1A1 is leading to the formation of the ultimately reactive species, 9-hydroxy-BaP-4,5-epoxide, that can react with deoxyguanosine in DNA (adduct 2; structure unknown). Insert: Autoradiographic profiles of BaP-DNA adducts, measured by ^{32}P-postlabelling, formed in the lungs of BaP-treated *Trp53(+/+)* mice (see also Supporting Figure 3A) or *ex vivo* in hepatic microsomes isolated from BaP-pretreated *Trp53(+/+)* mice (see also Figure 3); the origin, at the bottom left-hand corners, was cut off before exposure.

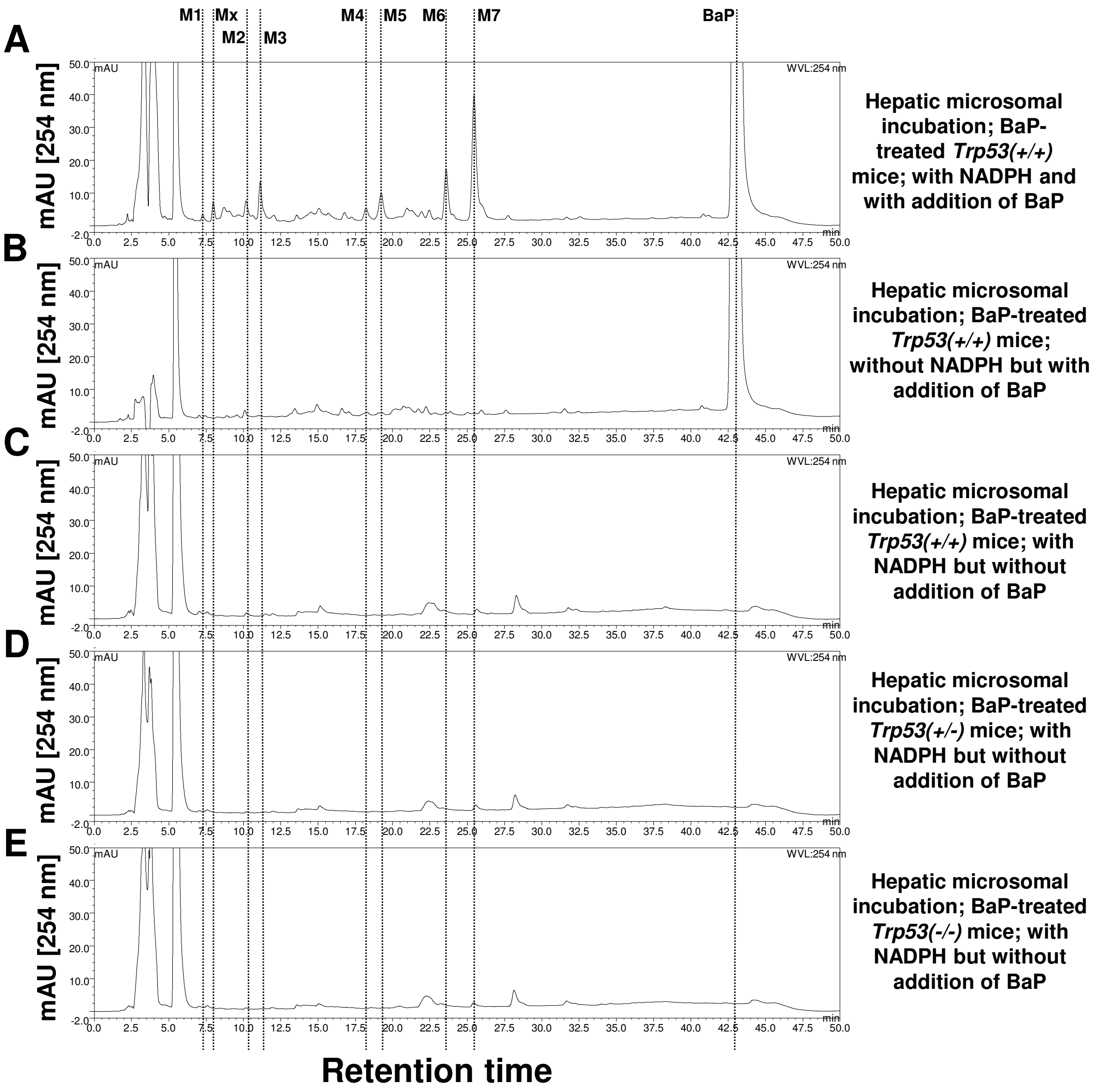

Supporting Figure 7

(A) Representative HPLC chromatogram of the BaP metabolites generated hepatic microsomal incubations of BaP-pretreated *Trp53(+/+)* with NADPH and BaP. (B) Representative HPLC chromatogram of the BaP metabolites generated hepatic microsomal incubations of BaP-pretreated *Trp53(+/+)* with BaP but without NADPH. Representative HPLC chromatogram of the BaP metabolites generated hepatic microsomal incubations of BaP-pretreated *Trp53(+/+)* (C), *Trp53(+/-)* (D) and *Trp53(-/-)* mice (E) without the addition of BaP.

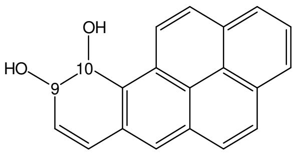

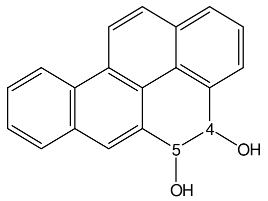

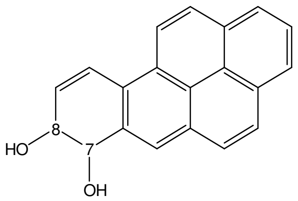

M1 = BaP-9,10-dihydrodiol **M2 = BaP-4,5-dihydrodiol** **M3 = BaP-7,8-dihydrodiol**

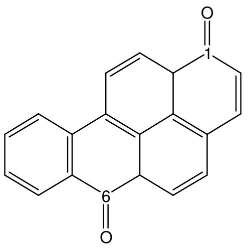

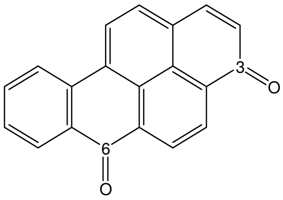

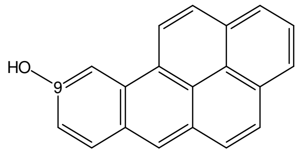

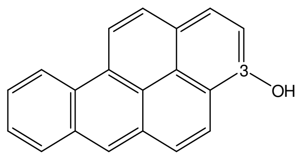

M4 = BaP-1,6-dione **M5 = BaP-3,6-dione** **M6 = BaP-9-ol** **M7 = BaP-3-ol**

Supporting Figure 8

Structures of BaP metabolites analysed by HPLC analysis.

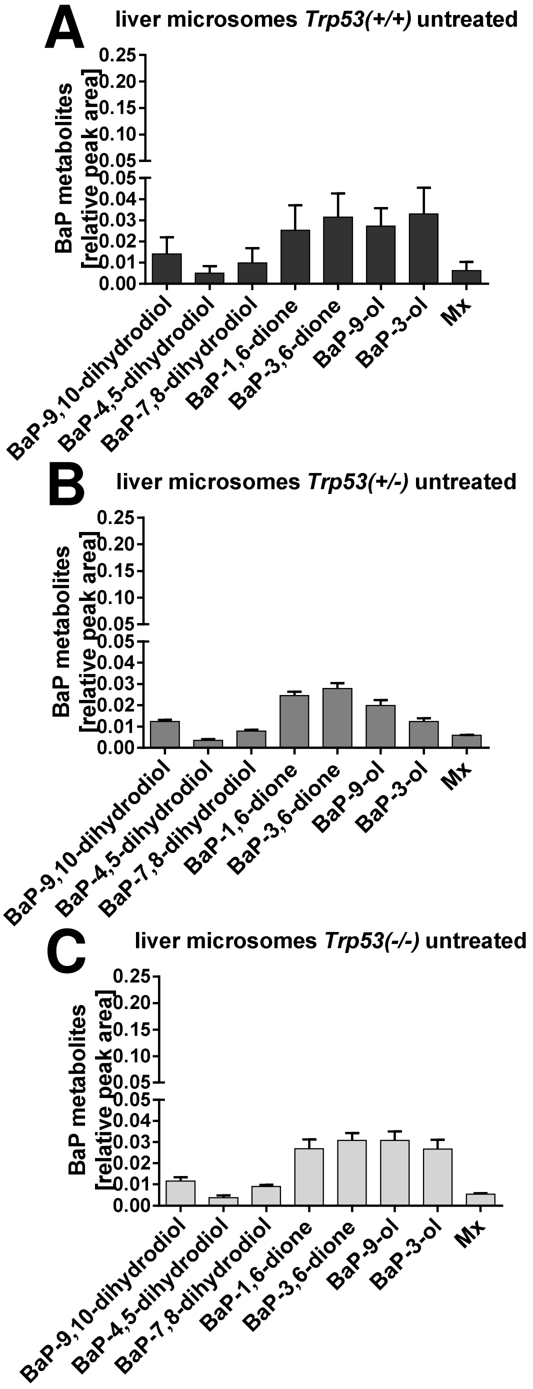

Supporting Figure 9

Formation of BaP metabolites by hepatic microsomes isolated from control (untreated) *Trp53(+/+)* (A), *Trp53(+/-)* (B) and *Trp53(-/-)* mice (C). Relative peak areas of BaP metabolites were measured by HPLC analysis at 254 nm. Values are the mean ± SD (n = 3). Statistical analysis was performed by one-way ANOVA followed by Tukey post-hoc test; no significant differences were observed. Structures of the BaP metabolites detected by HPLC are shown in Supplementary Figure 8. Mx, an unknown BaP metabolite.

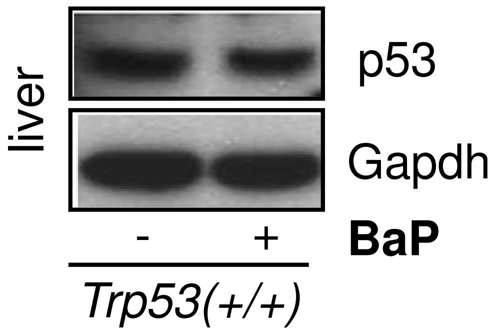

Supporting Figure 10

Western blot analysis of p53 in the livers of *Trp53(+/+)* mice exposed to BaP. Representative images of the Western blotting are shown; at least duplicate analysis was performed from independent experiments. Gapdh protein expression was used as loading control.

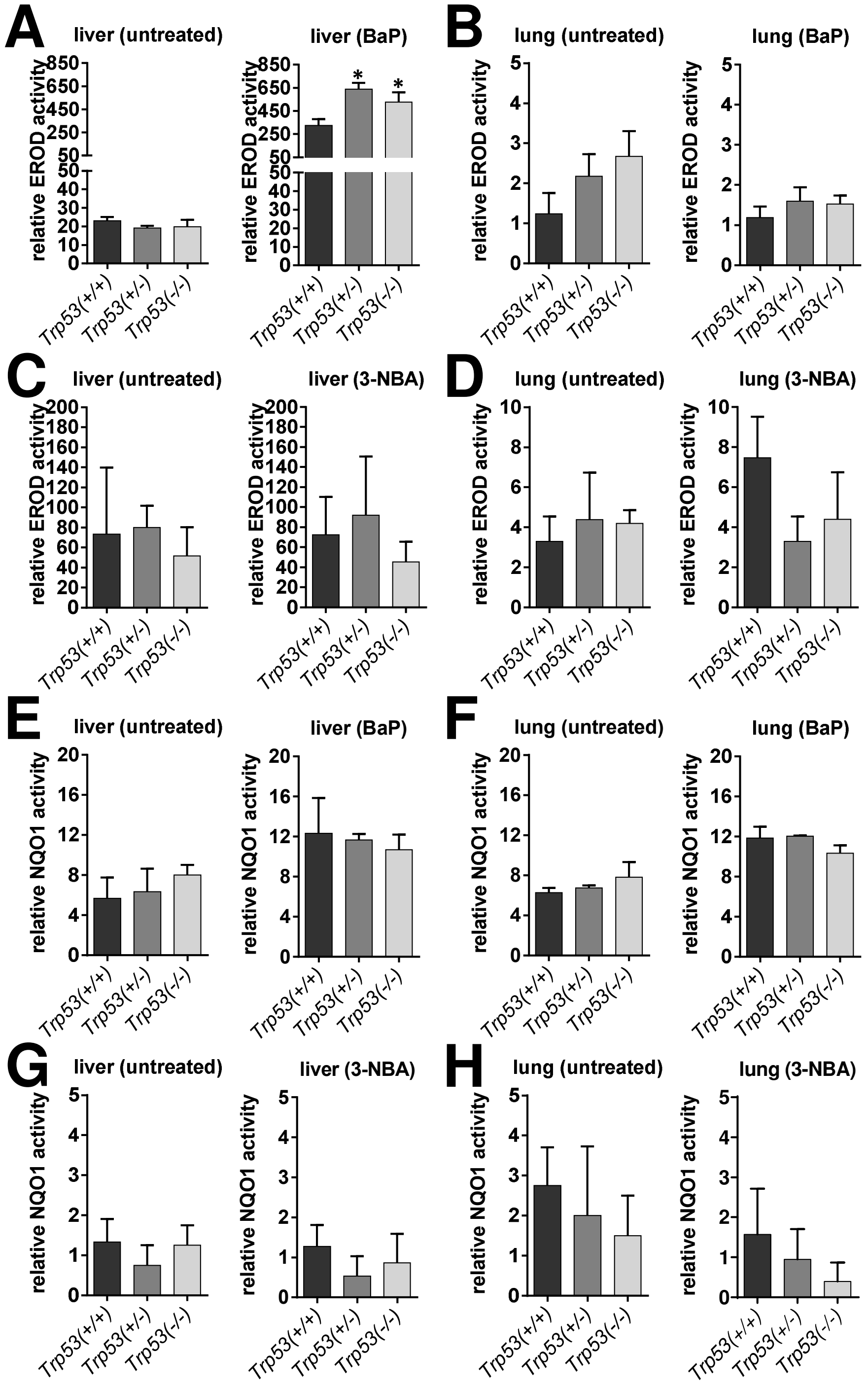

Supporting Figure 11

EROD activity (A-D) in hepatic (A and C) and pulmonary microsomes (B and D) isolated from *Trp53(+/+)*, *Trp53(+/-)* and *Trp53(-/-)* mice. Nqo1 enzyme activity (E-H) was determined in hepatic (E and G) and pulmonary cytosols (F and H). Values are the mean ± SD (n = 4). Statistical analysis was performed by one-way ANOVA followed by Tukey post-hoc test (*p<0.05; different from *Trp53(+/+)* mice).

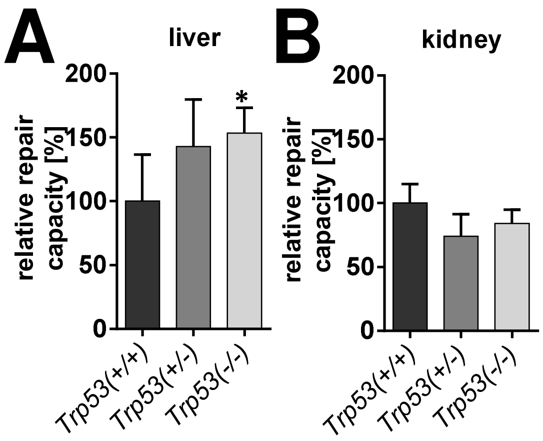

Supporting Figure 12

NER capacity in liver (A) and kidney (B) of *Trp53(+/+)*, *Trp53(+/-)* and *Trp53(-/-)* mice as assessed by the comet assay. Values are the mean ± SD (n = 4). Statistical analysis was performed by one-way ANOVA followed by Tukey post-hoc test (*p<0.05; different from *Trp53(+/+)* mice).

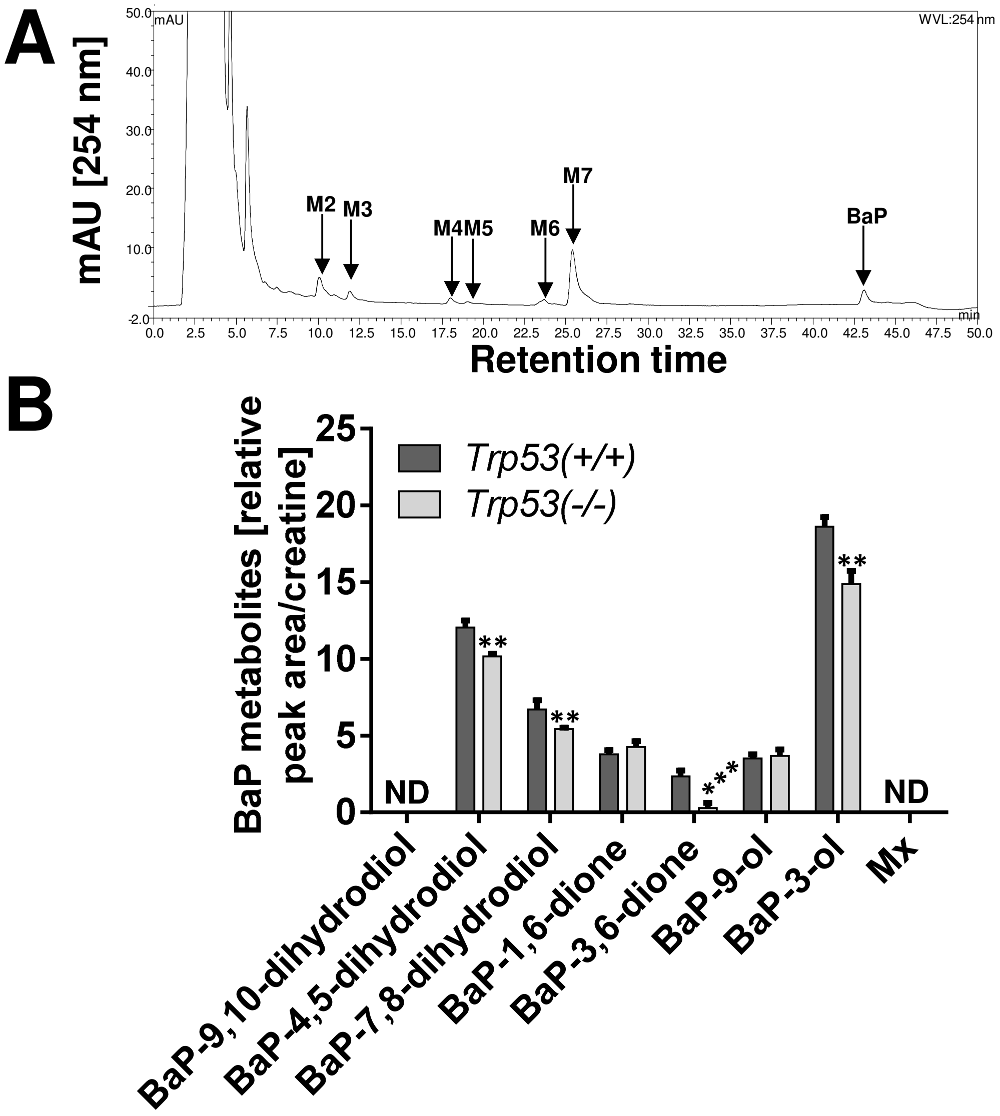

Supporting Figure 13

(A) Representative HPLC chromatogram of the BaP metabolites found in urine of *Trp53(+/+)* mice exposed to BaP. (B) BaP metabolites in urine of BaP-treated *Trp53(+/+)* and *Trp53(-/-)* mice. Relative peak areas of BaP metabolites were measured by HPLC analysis at 254 nm. Values are the mean ± SD (n = 3). Statistical analysis was performed by t-test analysis (**p<0.01, ***p<0.005; different from *Trp53(+/+)* mice). Mx, an unknown BaP metabolite. ND, not detected.
